# The Treatment of Lupus by X-Rays

**Published:** 1900-05-12

**Authors:** 


### THE TREATMENT OF LUPUS BY X-RAYS.

In view of the active, though injurious effects, which have from time to time been observed to result from the use of the *x* rays, and of the fact that these effects certainly extend far below the surface, it is not to be wondered at that many investigators have been led to endeavour to bring these powerful rays into some sort of use in the treatment of disease. It must be confessed that in our present state of ignorance as to the exact relation between the *x* rays and other manifestations of radiant energy it is almost waste of time to speculate as to the mode in which the *x* rays do good. Even in regard to the treatment of disease by radiant heat, or by radiant light as lately practised by Finsen, we can but guess as to the manner in which the curative effects are produced. What we do know about these proceedings seems to be that, whether it is a question of light, or heat, or *x* rays, or, maybe, of some form of electrical vibration emanating from the radiant points, the different tissues offer varying degrees of resistance to their transmission, and it is but reasonable to believe that where such resistance occurs it is accompanied by some tissue change, some phagocytosis, perhaps—temporary it may be, but sufficient to exert some influence upon morbid or lowly organised tissues, besides the directly germicidal influence which *x* rays, as well as direct light rays, may exert upon pathogenic microbes. Be this as it may, the evidence seems pretty clear that a very marked improvement is produced in cases of lupus by exposing the diseased patch to the full effect of the *x* rays. Dr. Eduard Schiff, of Vienna, claims to have been the first to cure lupus in this way, but since then the method has been tried in a fair number of cases. A remarkably good example of the successful use of the rays for this purpose is reported by Dr. R. E. Scholefield, in the *British Medical Journal* for May 5th. The nose was the part affected, and in using the rays the rest of the face was carefully protected. From the photographs given no one can doubt the beneficial effect of the applications.

